# Effect of 5-day dry immersion on the human foot morphology evaluated by computer plantography and soft tissues stiffness measuring

**DOI:** 10.1038/s41598-021-85771-9

**Published:** 2021-03-18

**Authors:** Alina Saveko, Liubov Amirova, Ivan Ermakov, Yuri Smirnov, Elena Tomilovskaya, Inesa Kozlovskaya

**Affiliations:** Russian Federation State Scientific Center—Institute of Biomedical Problems of the Russian Academy of Sciences, 123007, 76A Khoroshevskoe shosse, Moscow, Russia

**Keywords:** Bone quality and biomechanics, Neurophysiology, Sensorimotor processing, Sensory processing, Skeletal muscle

## Abstract

16 participants have been subjected to Dry Immersion model (DI) for 5 days. DI reproduces the space flight factors such as lack of support, mechanical and axial unloading, physical inactivity, elimination of vertical vascular gradient. Long-term bed rest is also associated with similar factors, so the results of the study may be useful for clinical medicine. Computer plantography and measuring the stiffness of the soft tissues of the foot and superficial muscles of the shin (mm. tibialis anterior and peroneus longus) were performed twice before DI exposure, on the 2nd and 4th days of DI exposure, as well as on the 2nd day of the recovery period. DI exposure effects the parameters under study in two ways: by raising the longitudinal arch and by flattening the transverse arch, which is accompanied by a decrease in the soft tissues stiffness of the foot and superficial muscles of the shin. The work reveals the phenomenon of compensating the longitudinal arch state by changing the characteristics that reflect the transverse arch state. The results of the study for the first time demonstrate the correlation of the foot morphological characteristics with a decrease in stiffness of mm. peroneus longus and tibialis anterior.

## Introduction

The effect of a 7-day space flight on foot morphology was examined in the Soviet-Cuban SUPPORT experiment. As a result, the first and only data on the effect of support-proprioceptive deprivation on the morphological characteristics of the foot were obtained by analyzing ink footprints on paper and X-ray images of the foot. The analysis of footprints after the space flight revealed a decrease in the area of the supporting surface in the region of the longitudinal arch, minor changes in the position of the toes, and transformation of the footprint in the metatarsal zone. At the same time, the X-ray results indicated that there are displacements of the talus, navicular, first and second cuneiform and first metatarsal bones, as well as “softening” of the ligamentous apparatus of the foot^1^. Only 2 cosmonauts participated in the experiment, which did not allow the authors to make final conclusions. Later on, such studies were conducted neither in space medicine nor in clinical medicine. However, these results justify the desirability of further studying the effect of hypokinesia and support unloading on the foot morphology.

The need for further research in this direction is confirmed by the fact that the foot is the first loaded segment, it provides contact with the supporting surface, strike absorption and load distribution along the overlying segments of the musculoskeletal system^2^. Moreover, it was proven that there is a correlation between morphological characteristics of the foot and a high risk of injuries of the musculoskeletal system: stress fractures of the bones of the foot, ankle, tibia and femur, various injuries of the soft tissues of the knee and ankle joint^3–8^.

Such a method of ground-based modeling of the effects of weightlessness factors on the human body as “dry” immersion (DI) can be used to investigate the effect of support-proprioceptive deprivation on the morphological characteristics of the foot. According to numerous studies, DI reproduces the following effects: a significant limitation of physical activity, support and weight axial unloading, as well as elimination of the vertical vascular gradient^9^. Given the qualitative similarity of acute and chronic responses to bed rest, “dry” immersion, and weightlessness^10^, studying the effect of DI on human foot morphology may be useful for practical medicine. Because the prolonged bed rest is directly related to hypokinesia in patients, support unloading, and forced unavoidable horizontal body position^11–13^.

We have suggested the hypothesis that the DI factors can elicit change in the foot morphology resulting from the reducing the transverse stiffness of soft tissues of the foot and muscles of the shin.

In view of the above, the aim of this work was to study the effect of 5-day “dry” immersion on the morphological characteristics of the foot using a computer scan of the supporting surface of the sole of the foot (computer plantography) with fixation of the support load, as well as to study the effect of DI factors on the stiffness of the soft tissues of the foot and superficial muscles of the shin (mm. tibialis anterior and peroneus longus).

## Methods

**Figure 1:**
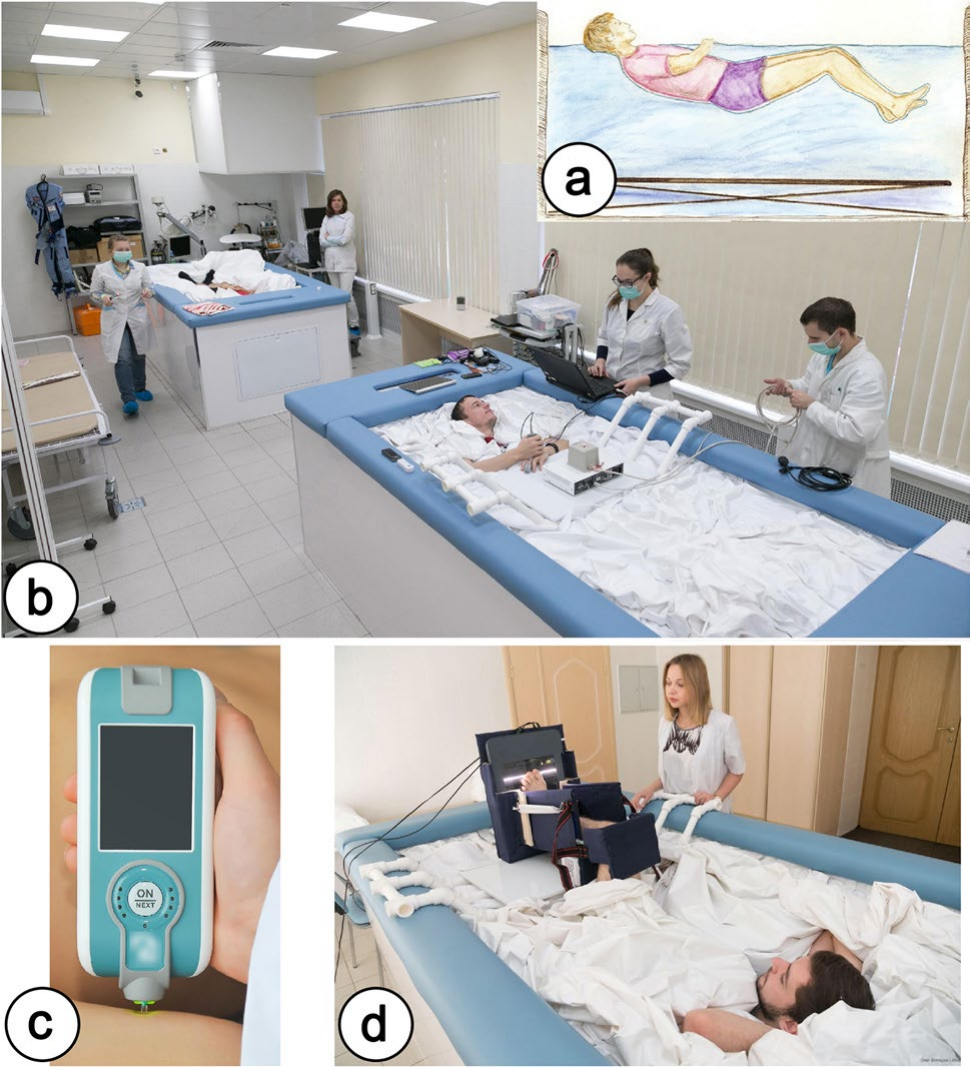
Methods used in the study. (**a**) Position of a participant in the immersion bath. (**b**) The facility “Dry Immersion”. (**c**) Appearance of the Myoton-Pro device (Myoton, Estonia). (**d**) Appearance of the computer plantography device.

The study was conducted at the Russian Federation State Scientific Center—Institute of Biomedical Problems of the Russian Academy of Sciences using the “Dry Immersion” facility which is a part of the “Medical and Technical Complex for the development of innovative space biomedicine technologies for the purpose of ensuring orbital and interplanetary flights, as well as for the development of practical healthcare” (Fig. 1b).

### Subjects

The study involved 16 healthy participants aged 33 ± 4.7 years (height: 173.1 ± 5.3 cm; weight: 73.6 ± 5.7 kg; body mass index: 24.5 ± 1.3 kg/m^2^). The participants were allowed to participate in the experiment by the medical expert commission of the Russian Federation State Scientific Center—Institute of Biomedical Problems of the Russian Academy of Sciences and signed an Informed Consent to participate in the study in accordance with the provisions of the Helsinki Declaration of Human Rights. Inclusion criteria: male, body mass index < 30 kg/m^2^, height ≤ 180 cm. Exclusion criteria: the presence of pathologies of the respiratory, digestive, urinary, sensorimotor, circulatory, nervous and integumentary systems that cause additional risks to health and life when exposed to DI factors. The research procedure was preliminarily reviewed and approved by the Biomedical Ethics Commission of the Russian Federation State Scientific Center—Institute of Biomedical Problems of the Russian Academy of Sciences. All the people represented on the pictures gave their informed consent for publication of identifying information/images in an online open-access publication.

### Conditions of dry immersion

Design of Dry Immersion was based on the previous research^9^. Dry Immersion is one of the most widely used ground-based models of microgravity. DI accurately and rapidly reproduces most of physiological effects of short-term space flight. The model simulates such factors of space flight as lack of support, mechanical and axial unloading, physical inactivity as well as elimination of the vertical vascular gradient. A test subject wearing a shirt and trunks was put on waterproof fabric and immersed into a deep bath up to the neck level, in a supine position. The area of the fabric’s surface considerably exceeded the area of the water surface. The folds of the waterproof fabric allowed the person’s body to be enveloped from all sides freely. Healthy male volunteers were placed individually in a supine position in a bath with dimensions of 200 × 100 × 100 cm. The bath was filled with water at a temperature that was kept constant at 33 ± 0.5 °C. The daily routine was specified in accordance with the schedule of studies, including 8 h of sleep, 3–4 meals, a medical supervision program and experimental studies. The research participants were taken out of DI for 15–20 min each day for sanitary and hygienic procedures with the usage of a special lift rising from the bottom of the bath (Fig. 1a).

The duration of DI exposure was 5 days. The baseline values were measured twice before exposure into the immersion bath. Then, the experimental sessions were conducted on the 2nd and 4th days of DI exposure, as well as on the 2nd day of the recovery period.

### Study of morphological characteristics of the foot

To obtain data on the morphological characteristics of the foot, we used the computer plantography method based on obtaining a graphic image of the supporting surface of the foot. For its implementation, we developed a special scanner design that allows scanning of the foot in a lying position in the immersion bath, eliminating the influence of position and body weight on the scan result. The special scanner design consisted of 2 parts: the flatbed scanner (scanner matrix type—CCD; lighting—a fluorescent lamp with a cold cathode) and the scanner case with solid elements to fix the position of the scanner and participant’s legs (90° in the knee and ankle joints) and tight elastic elements between the scanner and the knee joint to fix the magnitude of the supporting load on the surface of the scanner (Fig. 1d).

All foot scans, including baseline studies, were performed in a lying-down position in the immersion bath. The lower limb of the participant was at rest, while the knee and ankle joints were at 90° angles under control of the researcher. The magnitude of the supporting load on the surface of the scanner was the same in all experimental sessions and was equal to 15 kg owing to a special fixation system. The selected load of 15 kg is the maximum permissible load on the surface of the scanner and safe when using the solid elements to fix the position of the scanner and participant’s legs. At the same time, taking into account the mechanical properties of the foot^14^, this load is sufficient to initiate elastic deformation of the arches of the foot, which allows us to evaluate the morphofunctional characteristics of the foot based on the data obtained.

**Figure 2:**
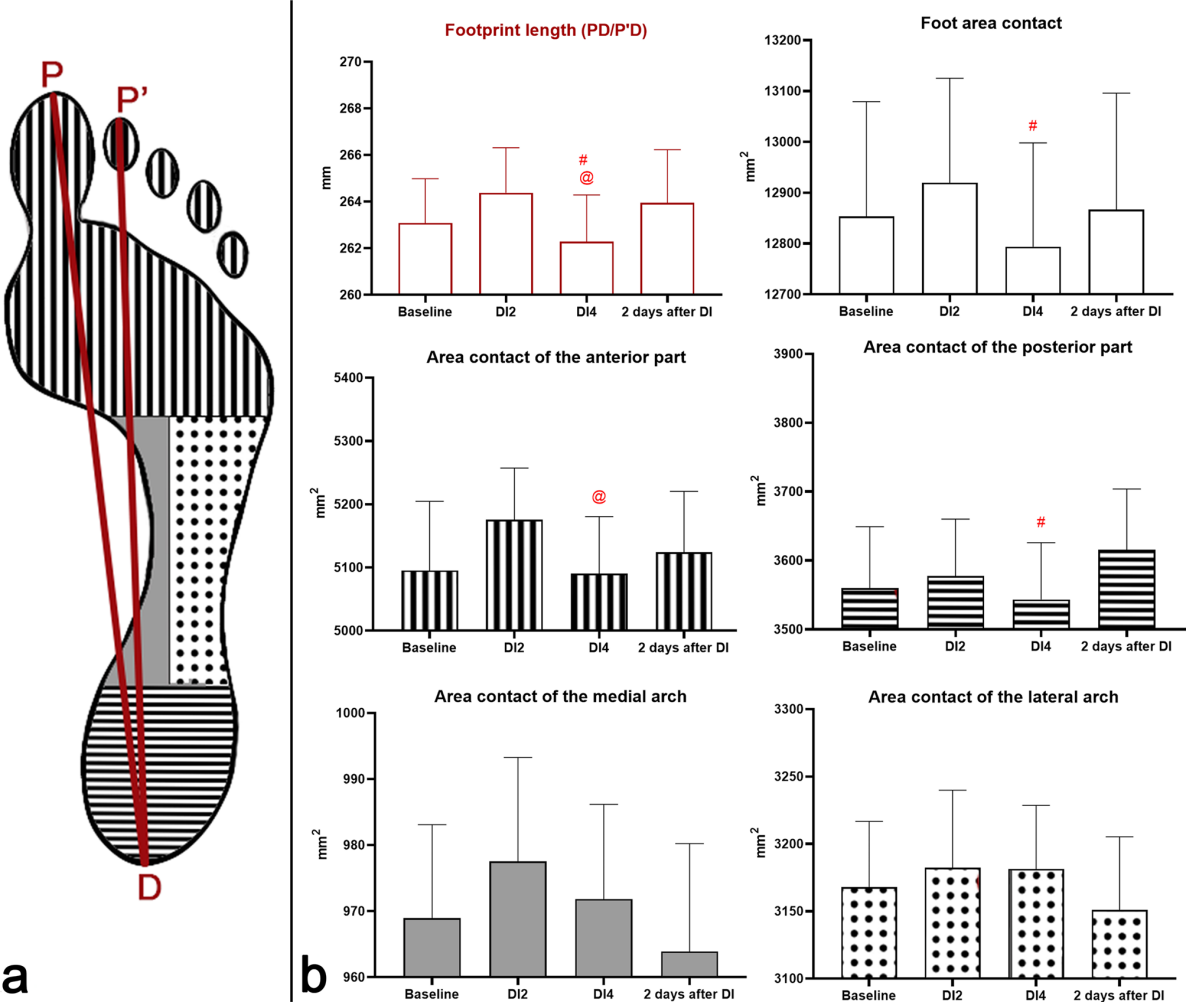
(**a**) Graphic image of morphological characteristics of the foot under study. (**b**) Changes in the length of the footprint (in mm), as well as contact areas of the foot (in mm^2^). On the abscissa axis, the experimental sessions are indicated: “Baseline”—the results obtained before “dry” immersion; “DI2”—the results obtained on the 2nd day of DI; “DI4”—the results obtained on the 4th day of DI; “2 days after DI”—the results obtained on the 2nd day after the completion of DI. *—reliable difference compared with the baseline values (p < 0.05). @—reliable difference compared with the values obtained on the 2nd day of DI (p < 0.05). #—reliable difference compared with the values obtained on the 2nd day after the completion of DI (p  <  0.05).

**Figure 3:**
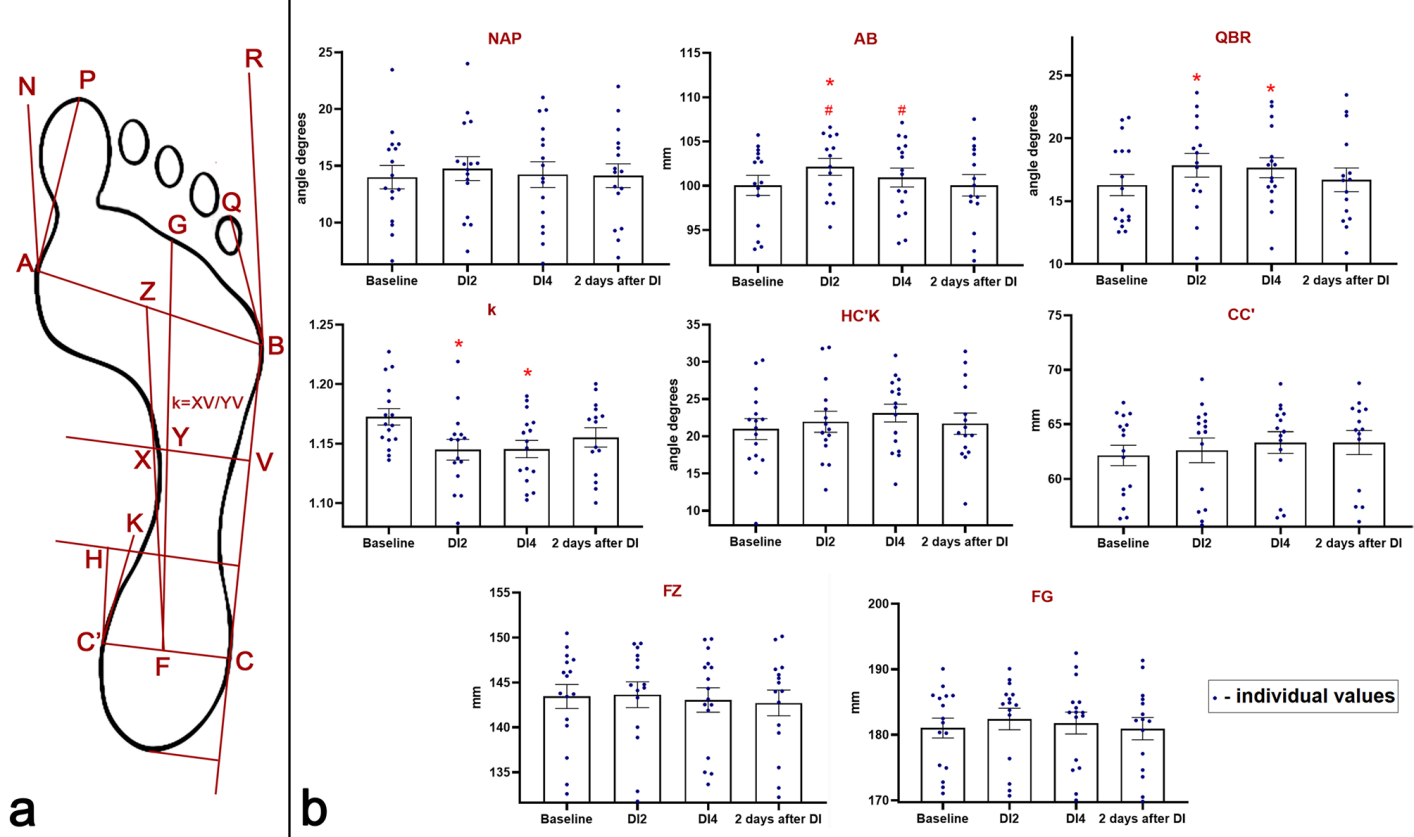
(**a**) Graphic image of morphological characteristics of the foot under study. (**b**) Changes in the lengths of the segments AB, CC’, FZ, FG (in mm), the angles NAP, QBR, HC’K (in degrees), as well as the coefficient k. On the abscissa axis, the experimental sessions are indicated: “Baseline”—the results obtained before “dry” immersion; “DI2”—the results obtained on the 2nd day of DI; “DI4”—the results obtained on the 4th day of DI; “2 days after DI”—the results obtained on the 2nd day after the completion of DI. *—reliable difference compared with the baseline values (p < 0.05). @—reliable difference compared with the values obtained on the 2nd day of DI (p < 0.05). #—reliable difference compared with the values obtained on the 2nd day after the completion of DI (p < 0.05).

The foot scanner was connected via a USB connector to a computer, where a graphic image of the supporting surface of the foot was recorded. The next stage of the graphic data processing was performed using a program that was specially written for this study; its algorithm is based on the method of measuring the morphofunctional characteristics of the foot provided in the Russian Federation patent No 2253363 (Gavrikov 2005). The algorithm for processing the graphical representation of footprints allows to estimate the length of the segments in mm, the angles in degrees and the flatfoot coefficient k. The measured characteristics are graphically shown in Figs. 2a and 3a. According to the method for measurement of the morphofunctional characteristics of the foot, the AB segment is the distance between the extreme points of the metatarsus on the side of the big toe (A) and the little toe (B). The CC′ segment is the distance between the extreme points of the heel on the side of the big toe (C′) and the little toe (C). The PD/P'D segment is the distance between the extreme posterior point of the footprint (D) and the extreme anterior point of the big toe (P) or the footprint (Pc. The longest of the PD and P′D segments characterizes the length of the footprint. The FZ segment is the distance between the midpoints of the AB segment (F) and CC′ segment (Z). The FG segment is the distance between the midpoint of the AB segment and the point between the base of the 3rd and 4th toes (G). The point E is the intersection point of a straight line passing through points BC and the ray from point D perpendicular to that line (E). On the straight line passing through points EB the point V is placed at the distance equal to 46% of the length of the footprint from the point E (V). The VV′ straight line is perpendicular to the EB straight line. The XV segment is the distance between the intersection point of the FZ segment with the VV′ straight line (X) and the point V. The YV segment is the distance between the intersection point of the FG segment with the straight line VV′ (Y) and the point V. The flatfoot coefficient k is equal to the ratio of the XV segment to the YV segment; it characterizes the state of the middle section of the longitudinal arch of the foot (k ≤ 0.5—the foot with an elevated longitudinal arch; 0.5 < k ≤ 1.10—the foot with a normal arch; 1.10 < k ≤ 1.20—the foot with a lowered longitudinal arch; 1.20 < k ≤ 1.30—the first degree of flatfoot; 1.30 < k ≤ 1.50—the second degree of flatfoot; k > 1.50—the third degree of flatfoot). On the straight line passing through points EB the point U is placed at the distance equal to 30% of the length of the footprint from the point E (U). The UU′ straight line is perpendicular to the EB straight line. The point H is the intersection point of the UU′ straight line and a ray from point C perpendicular to that line (H). The point K is the intersection point of the tangent line to the heel contour and the UU′ straight line (K). In turn, the heel angle HC′K characterizes the posterior part of the longitudinal arch of the foot (HC′K ≥ 5°—the foot with a normal arch; HC'K < 5°—the foot with a lowered longitudinal arch). The point N stands so that a straight line passing through points AN is parallel to the FZ straight line. The NAP angle characterizes the transverse arch from the medial side (NAP < 18°—the foot with a normal arch; NAP ≥ 18°—the foot with a lowered transverse arch). The point Q is the extreme anterior point of the little toe (Q). The point R stands so that a straight line passing through points BR is parallel to the FZ straight line. The QBR angle characterizes the transverse arch from the lateral side (QBR < 12°—the foot with a normal arch; QBR ≥ 12°—the foot with a lowered transverse arch). The method of measuring the morphofunctional characteristics of the foot has proven its reliability in a number of studies^15,16^.

During each experimental session, the right and left foot were scanned separately 3 times each; thus, one session consisted of 6 scans of the foot, the results of which were then averaged.

### Study of transverse stiffness of soft tissues of the foot and shin

To assess the transverse stiffness of soft tissues we used a Myoton-Pro device (Myoton, Estonia) which has proven its reliability in a number of studies^17–19^. The sensor of the Myoton-Pro device is a thin probe with a cross-sectional diameter of 3 mm; during the measurement, it is pressed by the researcher into the skin with a small force (0.18 N) perpendicular to the muscle under study (Fig. 1c).

When the required force is reached, the sensor of the device gives out five short (15 ms) mechanical pulses of stable force (0.4 N) with an interval of 1 s. As a result, the viscoelastic properties of soft tissues are calculated based on their response pulses which are then averaged. It should be noted that in this method an important role is played by the tension of the structures of the tissues under study.

In this study, we analyzed the parameter stiffness which is one of the main parameters characterizing viscoelastic properties of the myofascial complex; it is measured in N/m^19^. The viscoelastic properties of soft tissues were measured and calculated by the Myoton Pro device software. The mathematical model for the calculation is embedded in the device.

**Figure 4:**
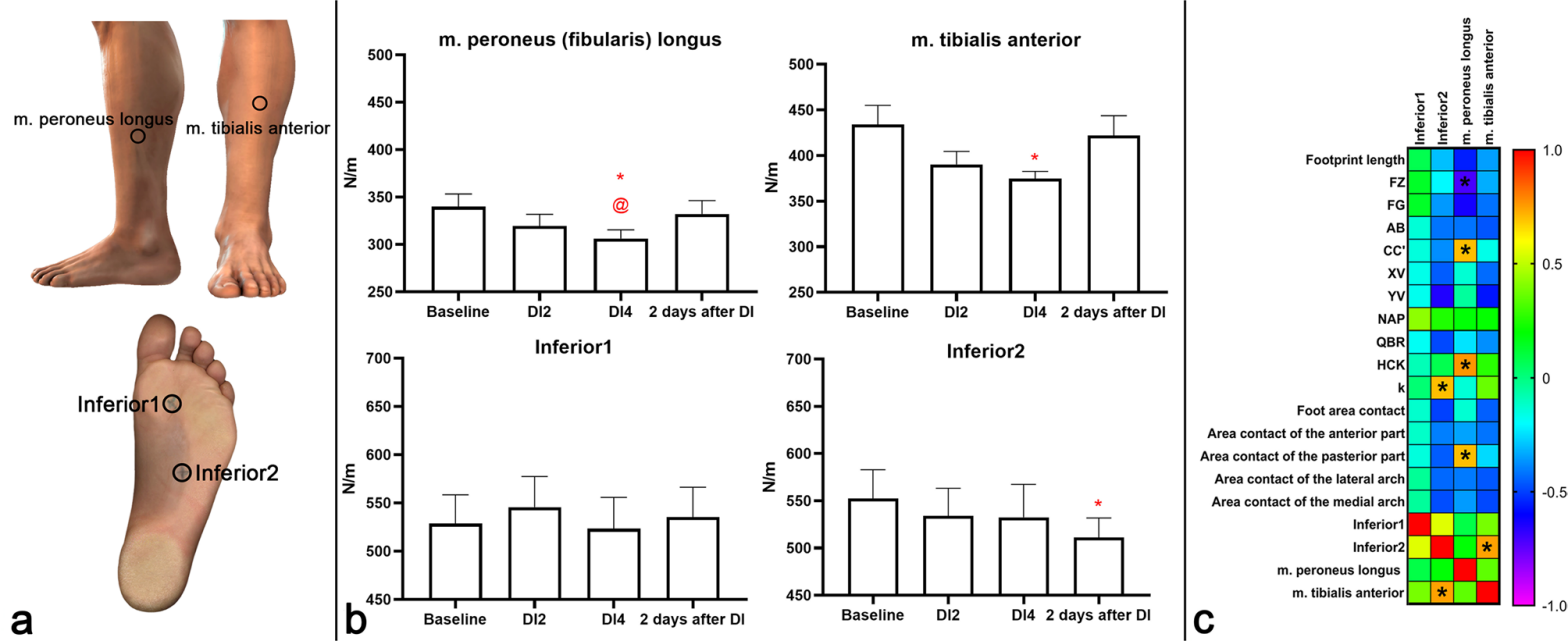
(**a**) Graphic image of places of measurement of the stiffness of soft tissues. (**b**) Changes in the stiffness of soft tissues at the points Inferior1 and Inferior2, as well as in projections of m. peroneus longus and m. tibialis anterior (in N/m). On the abscissa axis, the experimental sessions are indicated: “Baseline”—the results obtained before “dry” immersion; “DI2”—the results obtained on the 2nd day of DI; “DI4”—the results obtained on the 4th day of DI; “2 days after DI”—the results obtained on the 2nd day after the completion of DI. *—reliable difference compared with the baseline values (p < 0.05). @—reliable difference compared with the values obtained on the 2nd day of DI (p < 0.05). #—reliable difference compared with the values obtained on the 2nd day after the completion of DI (p < 0.05). (**c**)—Correlation matrix of morphological characteristics of the foot and parameters of the stiffness of soft tissues with a color indication of the degree of interrelation (p < 0.05). *—Pearson’s correlation coefficient r ≥  ± 0.70.

The measurements were made at 4 points indicated in Fig. 4a. Two points were located on the foot: the first one laterally from the head of the first metatarsal bone (Inferior1), the second one laterally from the junction of the first metatarsal bone with the medial cuneiform bone (Inferior2). Two points were located in the projection of two antagonist muscles: the first one in the projection of m. peroneus longus, the second one in the projection of m. tibialis anterior. These muscles were selected due to their functions and superficial location. The tendon of m. tibialis anterior is attached to the medial cuneiform bone and the base of the 1st metatarsal bone, due to which this muscle is involved in maintaining the medial longitudinal arch of the foot^20^. M. peroneus longus affects both the longitudinal and transverse arches of the foot^21,22^, since it follows the outer surface of the calcaneus and passes to the sole, where it obliquely crosses the foot and attaches to the tuberosity of the 1st and the base of the 2nd metatarsal bones.

All measurements, including baseline studies, were performed in the immersion bath in a lying-down/prone position. The subjects were relaxed and body segments were lying on soft surface of immersion bath thus limbs were slightly bent at the joints. The angle between the hip and shin was approximately 170 ± 10°, and the angle between the foot and the shin was also approximately 170 ± 10°.

### Data analysis

For data analysis, we used the GraphPad Prism version 8 (GraphPad Software, USA). The data were analyzed using repeated measures ANOVA. All significance levels were set at p ≤ 0.05. For assessment of relationship between two quantitative, continuous variables, we used the Pearson’s correlation coefficient (r). The values of r ≥  ± 0.70 were considered a strong correlation^23^. The data in the graphs are presented as mean with the standard error of the mean. The results of two baseline experimental sessions were averaged. The data were checked and distributed normally.

## Results

### Length and contact area of footprint

Before immersion in the immersion bath, the length of the footprint was 263.09 ± 7.61 mm (baseline). On the 2nd day of DI, this indicator increased by 0.49 ± 0.11%. Despite the tendency to increase in the first 2 days of DI, on the 4th day of DI the footprint length reliably decreased by 0.79 ± 0.20% (p < 0.05) compared with the 2nd day of DI, and it became by 0.31 ± 0.12% lower than the baseline values. On the 2nd day after the completion of DI, the values of the footprint length were reliably higher by 0.63 ± 0.14% (p < 0.05) than the values obtained on the 4th day of the DI, and also higher than the baseline values by 0.32 ± 0.17% (Fig. 2b).

The changes of the total area of contact of the foot with the surface of the scanner was similar to the changes of the length of the footprint. In baseline studies, the total contact area of the foot was 12,811.47 ± 858.45 mm^2^. On the 2nd day of DI, it increased by 0.84 ± 0.63%. Also on the 2nd day of DI, there was an increase in the contact areas of the forefoot (by 1.05 ± 0.54%), the medial arch (by 0.95 ± 0.42%), the posterior part of the foot (by 0.60 ± 0.37%), and the lateral arch (by 0.52 ± 0.21%).

On the 4th day of DI, the total contact area decreased by 0.97 ± 0.78% compared with the 2nd day of DI. An important role here was played by a change in the contact area of the forefoot—this indicator was reliably lower by 1.64 ± 0.23% (p < 0.05) than the values obtained on the 2nd day of DI. At the same time, the contact areas of the posterior part of the foot decreased by 0.95 ± 0.41%, the medial arch—by 0.58 ± 0.34%, and the lateral arch—by 0.03 ± 0.07%.

On the 2nd day after DI, the total foot contact area was reliably higher by 0.57 ± 0.24% (p < 0.05) than the values obtained on the 4th day and by 0.43 ± 0.37% higher than the baseline values. During the recovery period, the largest changes compared with the 4th day of DI occurred in the contact area of the posterior part of the foot which reliably increased by 2.03 ± 0.82% (p < 0.05), exceeding the baseline values by 1.67 ± 0.76%. At the same time, on the 2nd day after DI, the forefoot area increased by 0.66 ± 0.31% and exceeded the baseline values by 0.53 ± 0.43%, the area of the medial arch decreased by 0.81 ± 0.27% and became lower than the baseline values by 0.45 ± 0.43%, whereas the area of the lateral arch decreased by 0.96 ± 0.56% and became lower than the baseline values by 0.46 ± 0.54% (Fig. 2b).

### Parameters reflecting state of transverse arch of the foot

Before DI, the AB segment was equal to 100.04 ± 4.37 mm. The NAP angle was equal to 13.99 ± 3.89° and corresponded to the foot with a normal transverse arch, only 1 participant had the value of this angle that was characteristic of the foot with a lowered transverse arch. In the baseline studies, all participants had the values of the QBR angle that corresponded to the foot with a lowered transverse arch and were equal to 16.28 ± 3.36°.

On the 2nd day of DI, a lowering of the transverse arch of the foot was indicated by a change in the following characteristics: a reliable increase compared to the baseline values in the AB segment by 2.09 ± 0.86% (p < 0.05), in the QBR angle by 9.65 ± 2.07% (p < 0.05), and in the NAP angle by 0.99 ± 0.57%.

On the 4th day of DI, an inversion of the trends of characteristics compared with the 2nd day of DI took place: a decrease in the AB segment by 1.17 ± 0.15% and a decrease in the QBR angle by 1.10 ± 0.21%. It should be noted that despite the decrease these indicators remained higher than the baseline values by 0.88 ± 0.31% and 8.44 ± 3.15% (p < 0.05), respectively. However, the NAP angle increased by 0.65 ± 0.55% compared with the 2nd day of DI and became higher than the baseline values by 1.65 ± 1.03%.

2 days after DI, the AB segment reliably decreased by 0.87 ± 0.11% (p < 0.05) compared with the 4th day of DI and reached a reliable difference with the values on the 2nd day of DI, returning to the baseline values. At the same time, the values of the QBR angle also decreased by 5.44 ± 2.14% compared with the 4th day of DI, but remained higher than the baseline values by 2.54 ± 1.32%. The NAP angle was practically unchanged compared to the 4th day of DI and remained higher than the baseline values by 1.50 ± 0.72%.

It is interesting that one of the participants before DI had the QBR angle corresponding to the foot with a lowered transverse arch, but on the 2nd day of DI his QBR angle decreased and began to correspond to the foot with a normal transverse arch until the end of the experiment. Also, on the 2nd day of DI, the values of the NAP angle in three participants increased so much that they began to correspond to the foot with a lowered transverse arch until the end of the experiment, and only in one of the three participants this indicator returned to the normal values on the 2nd day after DI (Fig. 3b).

### Parameters reflecting state of longitudinal arch of the foot

Before the experiment, the coefficient k reflecting the state of the longitudinal arch was equal to 1.173 ± 0.027. In three participants, this indicator was higher than 1.20 and corresponded to the 1st degree flatfoot, in the other participants it was higher than 1.10 and corresponded to the foot with a lowered longitudinal arch. The baseline values of the heel angle HC′K corresponded to the norm and were equal to 20.97 ± 5.54°.

On the 2nd day of DI, the longitudinal arch of the foot became higher, as indicated by a reliable decrease in the coefficient k by 2.32 ± 0.97% (p < 0.05) and an increase in the heel angle HC′K by 4.61 ± 3.23%.

On the 4th day of DI, the coefficient k did not change compared with the 2nd day of DI, whereas the HC′K angle continued to increase by 5.30 ± 4.54%.

On the 2nd day after the completion of DI, the longitudinal arch had a tendency to decrease, which is indicated by an increase in the coefficient k by 0.84 ± 0.23% and a decrease in the HC′K angle by 6.1 ± 4.71% compared with the 4th day of DI. At the same time, the coefficient k remained below the baseline values by 1.47 ± 0.34%, while the HC′K angle became higher by 3.43 ± 1.47%.

It should be noted that in all three participants who had the 1st degree flatfoot before DI, the values of the coefficient k decreased during the experiment to those characteristic of a lowered longitudinal arch of the foot: in two—on the 2nd day of DI, and in one—on the 4th day of DI. Also, on the 2nd day of DI in one participant with an initially lowered longitudinal arch, the coefficient k reached the values characteristic of a normal longitudinal arch of the foot and remained at this level until the end of the experiment (Fig. 3b).

### Transverse stiffness of soft tissues of the foot and shin

The stiffness of soft tissues before DI at the point Inferior1 was equal to 528.65 ± 73.22 N/m, at the point Inferior2—552.55 ± 74.25 N/m, in m. peroneus longus—339.91 ± 48.39 N/m, in m. tibialis anterior—433.96 ± 78.71 N/m.

On the 2nd day of DI, an increase in the stiffness was observed only at the Inferior1 point—by 3.24 ± 0.77%, while at the other points of measurement the stiffness decreased: in m. tibialis anterior by 10.07 ± 1.15%, in m. peroneus longus by 5.99 ± 1.35%, at the Inferior2 point by 3.35 ± 2.02%.

On the 4th day of DI, all stiffness indicators were decreased. In m. tibialis anterior, the stiffness was decreased by 3.99 ± 0.83%, as a result of which a reliable difference was achieved with the baseline values of 13.67 ±3.14% (p < 0.05). The stiffness in m. peroneus longus also reliably decreased by 4.10±1.11% (p < 0.05) compared with the 2nd day of DI and by 9.85 ± 3.97% (p < 0.05) compared with the baseline values. On the 4th day of DI, the stiffness at the Inferior1 point decreased by 4.11 ± 2.01% compared with the 2nd day of DI, and at the Inferior2 point by 0.23 ± 0.09%.

On the 2nd day after the completion of DI, the stiffness indicators at the Inferior1 point and in m. tibialis anterior and m. peroneus longus showed a tendency to recover, however, the stiffness at the Inferior1 point remained higher than the baseline values by 1.27 ± 0.56%, while the stiffness in m. tibialis anterior and m. peroneus longus was lower by 2.78 ± 0.35% and 2.25 ± 0.21%, respectively. Only at the Inferior2 point the stiffness indicators on the 2nd day after DI did not tend to recover, but decreased by 3.97 ± 0.54% compared to the 4th day of DI, reaching a reliable difference with the baseline values by 7.45 ± 0.48% (p < 0.05) (Fig. 4b).

### Correlation between parameters under study

**Figure 5:**
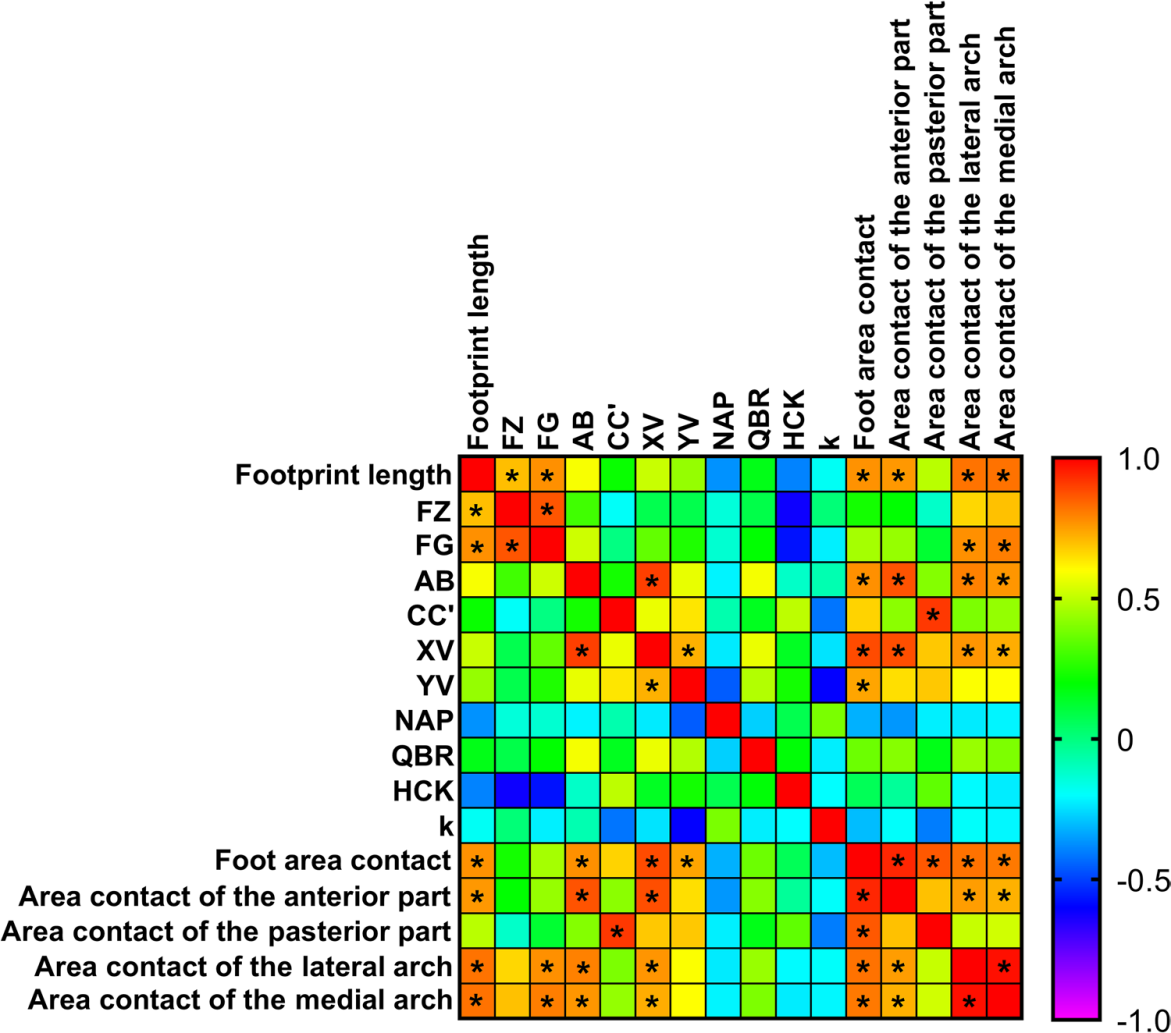
Correlation matrix of morphological characteristics of the foot between each other with a color indication of the degree of interrelation. *Pearson’s correlation coefficient r ≥  ± 0.70.

An analysis of the interrelations between the parameters under study during the entire experiment revealed that the length of the footprint and the segments AB and XV have a direct correlation (r ≥ 0.70) with all indicators of the contact area of the foot, except for the contact area of the posterior part of the foot (Fig. 5). The segments AB and XV correlated with each other (r = 0.90) and most depended on the contact area of the forefoot (r > 0.86). Also, the total contact area of the foot most depended on the contact area of the forefoot (r = 0.94). In turn, the forefoot area had a direct correlation with the areas of the medial and lateral arches of the foot (r = 0.72 and r = 0.75, respectively). The area of the posterior part of the foot correlated with the length of the CC′ segment (r = 0.91). As expected, between the segments XV and XY, as well as between the segments FG and FZ, a high correlation was found (r = 0.72 and r = 0.87, respectively). However, the XY segment, unlike the XV segment, depended only on the total contact area of the foot (r = 0.74). The FZ segment directly depended on the length of the footprint (r = 0.70), and the FG segment depended on the areas of the medial and lateral arches of the foot (r = 0.80 and r = 0.77, respectively) (Fig. 5).

A correlation analysis of the stiffness of soft tissues in the projection of m. peroneus longus revealed a direct correlation with the CC′ segment (r = 0.70), the HC′K angle (r = 0.76) and the contact area of the posterior part of the foot (r = 0.71), as well as an inverse correlation with the FZ segment (r = − 0.71). In turn, a correlation analysis of the stiffness of soft tissues at the Inferior2 point revealed a direct correlation with the coefficient k (r = 0.70) and the stiffness of soft tissues in the projection of m. tibialis anterior (r = 0.75) (Fig. 4c).

## Discussion

The obtained results allow to conclude that DI factors affect the morphological characteristics of the foot in two directions: raising the longitudinal arch and flattening the transverse arch of the foot. Note that the influence of these factors on the longitudinal arch of the foot was more pronounced. Based on the analysis of the changes in the studied parameters, we suggest that by the 4th day of immersion exposure those morphological changes that were recorded on the 2nd day of DI become compensated. This phenomenon was most pronounced in the characteristics reflecting the state of the forefoot and the transverse arch of the foot. We assume that this helps to prevent a further decrease in the transverse stiffness of the longitudinal arch (stiffness at the Inferior2 point) and in the coefficient k on the 4th day of DI. This assumption is supported by the correlation of the parameters of the forefoot (AB segment, contact area of the forefoot) and the parameters of the longitudinal arch of the foot (XV segment, contact areas of the medial and lateral arches) and by the previously studies. Venkadesan et al. have previously shown that the transverse tarsal arch, acting through the inter-metatarsal tissues, is responsible for more than 40% of the longitudinal stiffness of the foot^14^. Also in the Soviet-Cuban SUPPORT experiment using X-ray of the foot the upward displacement and pronation of the talus have been shown, resulting in the downward displacement of the navicular bone, what caused the downward displacement of the first and second cuneiform, as well as the first metatarsal bones. The authors associated these changes with the raising the longitudinal arch and flattening of the transverse arch of the foot^1^.

**Figure 6:**
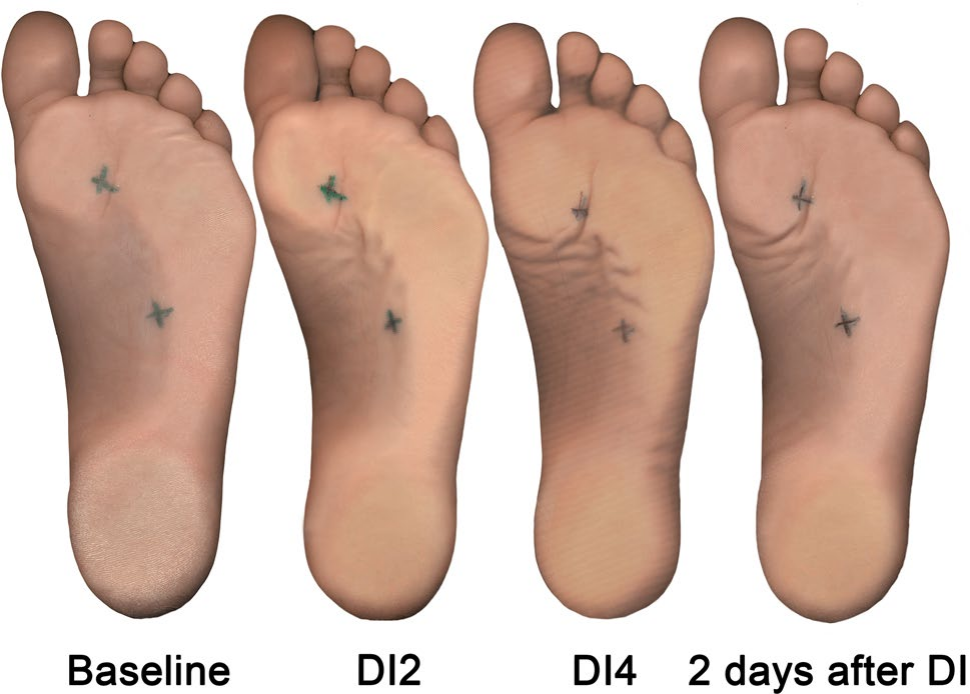
Demonstration of footprints obtained by scanning during an experiment in one participant. “Baseline”—the image obtained before “dry” immersion; “DI2”—the image obtained on the 2nd day of DI; “DI4”—the image obtained on the 4th day of DI; “2 days after DI”—the image obtained on the 2nd day after the completion of DI.

Illustrative example of changes of the footprints obtained by scanning during an experiment in one participant is shown in Fig. 6.

Also, the results of this study for the first time demonstrate the interrelation of the morphological characteristics of the foot with transverse stiffness of m. peroneus longus and m. tibialis anterior under conditions of DI.

This conclusion is consistent with the anatomy and function of these muscles. The m. tibialis anterior is the strongest dorsiflexor of the foot. Dorsiflexion is critical to gait because this movement clears the foot off the ground during the swing phase. The m. tibialis anterior, along with the m. tibialis posterior, is also a primary inverter of the foot. Because it arises from the lateral tibia and the tendon inserts on the medial border of the foot, muscle contraction lifts structures of the medial arch (medial cuneiform, first metatarsal, navicular, talus) into adduction-supination or inversion. The movement of inversion occurs at 2 synovial joints in the foot: the subtalar joint, between the talus and calcaneus, and the midtarsal joint, between the talus and navicular bone. The m. tibialis anterior, due to its insertion on the medial foot, also supports the medial longitudinal arch of the foot^20^. The m. peroneus longus muscle arises from the head and proximal and lateral side of the fibula and inserts on the base of the first metatarsal and medial cuneiform. The main function of the m. peroneus longus is to plantarflex and evert the foot at the ankle. Due to its insertion on the medial aspect of the foot and course down the lateral part of the leg, muscle contraction lifts the foot upward (plantar flexion) and outward (eversion). This movement is critical to eversion of the foot and can be injured commonly from forced inversion and dorsiflexion of the ankle in the setting of trauma^21^. In addition, Kokubo et al. have previously shown that the traction on the m. peroneus longus tendon decreased the stiffness of the foot^22^.

However, the question of the trigger mechanism for changes in both the morphological characteristics of the foot and the stiffness of the studied muscles during DI exposure remains open. Based on previous research, we assume that a significant role in reducing the transverse stiffness of these muscles played by the changes of support afferentation signal input^24^.

In turn, the morphology of the foot has a significant effect on the ratio between the forces of support reactions and the axes of rotation of the ankle and knee joints^3^. In 1987, Giladi et al. found that individuals with an increased longitudinal arch have a higher incidence of stress fractures of the tibia, femur and foot^4^. A similar relationship was found in the study of Cowan et al.^5^. Moreover, Dahle et al. associated pain in the knee with the structural features of the foot^6^, whereas Kaufman et al. noted that both increased and decreased longitudinal arch of the foot may contribute to an increased incidence of stress fractures^7^. This question was studied in more detail in 2001 by Williams et al. They showed that people with an increased longitudinal arch of the foot were more often affected by stress fractures of the foot and ankle, while the most common diseases in this group were plantar fasciitis, lateral sprains of the ankle joint, and iliotibial tract syndrome, and only the fifth metatarsal bone withstood stress fractures. People with a lowered longitudinal arch of the foot more often suffered from injuries of soft tissues of the knee, general knee pain, patellar tendonitis and plantar fasciitis, while the second and third metatarsal bones had metatarsal stress fractures^8^.

Due to the above and the fact that prolonged bed rest is directly related to hypokinesia in patients, support unloading and forced unavoidable horizontal body position^11–13^, it is worth noting that the results of the study may be useful for clinical medicine.

Thus, the present work helps to understand the mechanisms of the influence of DI factors on the morphological characteristics of the foot and the stiffness of the soft tissues of the foot and shin, as well as draws attention to the need for further research in this direction for advancement of both clinical and space medicine.
